# Differential Effects of Self- vs. External-Regulation on Learning Approaches, Academic Achievement, and Satisfaction in Undergraduate Students

**DOI:** 10.3389/fpsyg.2020.543884

**Published:** 2020-10-07

**Authors:** Jesús de la Fuente, Paul Sander, Douglas F. Kauffman, Meryem Yilmaz Soylu

**Affiliations:** ^1^School of Education and Psychology, University of Navarra, Pamplona, Spain; ^2^School of Psychology, University of Almería, Almería, Spain; ^3^School of Psychology, Teesside University, Middlesborough, United Kingdom; ^4^Medical University of the Americas − Nevis, Devens, MA, United States; ^5^University of Nebraska-Lincoln, Lincoln, NE, United States

**Keywords:** undergraduate students, satisfaction, academic achievement, learning approaches, SRL vs. ERL theory

## Abstract

The aim of this research was to determine the degree to which undergraduate students’ learning approach, academic achievement and satisfaction were determined by the combination of an intrapersonal factor (self-regulation) and a interpersonal factor (contextual or regulatory teaching). The hypothesis proposed that greater combined regulation (internal and external) would be accompanied by more of a deep approach to learning, more satisfaction and higher achievement, while a lower level of combined regulation would determine a surface approach, less satisfaction and lower achievement. Within an *ex post* facto design by selection, 1036 university students completed validated questionnaires using an online tool. Several multivariate analyses were conducted. Results showed that the combination of self-regulation and external regulation can be ordered as levels along a five-point scale or heuristic. These levels linearly determine type of learning approach, academic achievement and satisfaction. Implications are established for quality and improvement of the teaching and learning process at university.

## Introduction

The analysis of learning approaches, academic achievement and satisfaction at university, as well as their predictive factors, has been a constant in recent research in Educational Psychology (Balloo et al., 2017; Barattucci, 2017). Every university wants its students to experience good learning processes and attain high achievement and satisfaction with the educational experience; these matters impact institutional prestige and social desirability, not to mention their frequent use as criteria for assessing teaching quality (Browne et al., 1998; Elassy, 2015). Moreover, the degree of perceived satisfaction with the university forms part of the rankings that are published annually in many national and international listings (Douglas et al., 2015).

For all of the above, the choice of one model or another to explain academic achievement and the role of learning approaches is highly important in the practice of Educational Psychology at university (Green, 2014; Hazan and Miller, 2017). The present research study seeks to offer an alternative conceptual view, as well as empirical evidence to contribute to an integrated analysis of learning approaches, achievement and academic satisfaction, considering these as variables that depend on both learning and teaching processes in the formal university context (Biggs, 2001; Biggs and Tang, 2011; Barattucci et al., 2017; Kember et al., 2020).

## Self-Regulation (SR) and Regulatory Teaching (RT) as Variables of the Teaching and Learning Process: A Heuristic for Analysis

*Self-Regulation* (SR) has been defined as an intrapersonal (individual) variable that allows people to manage their decisions, making it possible for them to plan, exercise control over such decisions, and evaluate their effects (Brown, 1998). In psychology research on health and academic well-being, SR has been considered a variable at the molecular level (de la Fuente et al., 2019a). It is predictive of various specific regulatory behaviors, such as coping strategies (de la Fuente et al., 2019b) or achievement emotions (de la Fuente et al., 2020b, c). In the realm of educational psychology, it has been conceptualized as a meta-behavioral, student variable (presage), predictive of Self-Regulated Learning (process variable), achievement and academic satisfaction (product variables). Previous research has consistently established these relationships (Dinsmore et al., 2008; Kaplan, 2008; Antonelli et al., 2020). Thus, self-regulation (SR) as a personal variable may be considered a precursor to Self-Regulated Learning (SRL) (de la Fuente et al., 2008, 2015b).

*Regulatory teaching* (RT) has been defined as a contextual variable, referring to the degree to which the teaching process promotes and externally favors students’ SRL. It has been conceptualized as a meta-instructional variable; regulatory teaching encourages self-regulation in students and is characteristic of *effective teaching*. There have been many approaches to effective teaching in the research (for a review, see Goe et al., 2008; Baeten et al., 2013; Karagiannopoulou and Milienos, 2015). Empirical research identifies high quality teachers as those who positively influence their students’ engagement with learning activities, as well as students’ performance in learning (self-regulation, social competencies, academic achievement). Mediating factors in student performance must be considered (Roehrig and Chistesen, 2010): (1) Organization of the content and activities; (2) Planning for the majority of the class; (3) Encouraging deep processing and self-regulation. Recent research has shown that variables of the perceived classroom learning environment were good predictors of students’ self-regulation. Moreover, teacher variables (effective teaching) were found to be directly related to students’ self-regulation, and there were moderate relationships between learning environment and self-regulation variables (Kossak, 2019; Yerdelen and Sungur, 2019).

The theory of *Self-Regulated vs. Externally Regulated Learning, SRL vs. ERL* (de la Fuente, 2017) has attempted to identify and organize the different real-life combinations that result from the interaction between different types of university students and teachers (Azevedo et al., 2008). Specifically, this theory suggests that during any teaching-learning process, we find different levels of student self-regulation (low-medium-high) in combination with different levels (low-medium-high) of regulatory teaching. Consequently, a heuristic with five possible combination ranks has been put forward (see Table 1). This heuristic of combinations has been successfully evaluated in reference to the effect of its regulation levels on university students’ achievement emotions (de la Fuente et al., 2019b) and their coping strategies (de la Fuente et al., 2020b). However, its effect on learning approaches, satisfaction and achievement has yet to be reported, and this is the aim of the present research study.

**TABLE 1:** Heuristic of five combinations of the *Utility Model*^TM^ hypothesized by *SRL* vs. *ERL Theory* (de la Fuente, 2017).

*Combination Level*		*Regulation*	*Regulation Trend*	*Learning* Approaches*		*Academic.*	*Satisfact.*
SR Level (range)	RT Level (range)	*aver/rank*		Deep	Surface	Achiev.*	T & L*
**3** (3.85−5.00)**H**	**3** (2.84−5.00) **H**	3.0/**5**	**High-High:** *High-Regulation*	++	*–*	*H*	*H*
**2** (3.10−3.84)**M**	**3** (2.84−5.00) **H**	2.5/**4**	**Medium-High**: *Regulation*	+	*–*	*M-H*	*M-H*
**3** (3.85−5.00)**H**	**2** (2.35−2.83) **M**	2.5**/4**	**High-Medium**: *Regulation*	+	*–*	*M-H*	*M-H*
**2** (3−10−3.84)**M**	**2** (2.35−2.83) **M**	2.0/**3**	**Medium:** *Non-Regulation*	=	=	*M*	*M*
**2** (3.10−3.84)**M**	**1** (1.00−2.34) **L**	1.5/**2**	**Medium-Low**: *Dys-Regulation*	*–*	+	*M-L*	*M-L*
**1** (1.00−3.09) **L**	**2** (2.35−2.83) **M**	1.5/**2**	**Low-Medium**: *Dys-Regulation*	*–*	+	*M-L*	*M-L*
**1** (1.00−3.09) **L**	**1** (1.00−2.34) **L**	1.0/**1**	**Low-Low:** *High Dys-Regulation*	*–*	++	*L*	L

SR Level, Self-Regulation level (1−3 range); RT Level, Regulatory Teaching level (1−3 range); H, High level; M, Medium level; L, Low level; ++high amount of this type of learning approach; – low amount of this type of learning approach; =, medium amount of this type of learning approach; Academic Achiev., Academic achievement; Satisfact. T & L, Satisfaction with Teaching and Learning process. * Effects analyzed in this investigation. Please see and analyze the differences with previously research reports (de la Fuente et al., 2019b, p. 12; de la Fuente et al., 2020a, p. 5).

The Vermunt model (Vermunt, 1998; Vermunt and van Rijswijk, 1988), similar in part to SRL vs. ERL Theory (op cit., 2017), distinguished between three different strategies of regulation: self-regulation, external regulation and lack of regulation of learning (Lindblom-Ylänne et al., 2011):

(1) *Self-Regulated Learning*: referring to what students do to plan and monitor their learning activities, diagnose the cause of any problems that occur while learning, and progress toward the learning goals they have set for themselves. This definition is similar to other definitions or theories of learning, such as Winne (1995), Winne and Hadwin (1997), or Zimmerman (1998, 2000), who defined self-regulated learning as the systematic effort to direct one’s thoughts, feelings and actions toward meeting academic goals. Biggs (1985) used the term meta-learning to describe the state of being aware of and exercising self-control over one’s own learning.

Self-Regulated Learning vs. ERL Theory (de la Fuente, 2017) uses an identical concept of SRL, while also assuming that a prior variable (SR) may be what determines the level of SRL during learning. Self-regulated learning is assumed to be present at three levels: adequate, non-existent and low.

(2) *External Regulation of Learning*: External regulation refers to situations where students depend on a teacher’s guidance and control (or a text book, or classmates) to regulate learning processes. In this model, the teacher takes on the regulatory activities of the students.

In SRL vs. ERL Theory (2017), however, external regulatory actions are designed to assist and promote students’ internal self-regulation–not to exercise external control over them. As such, this type of external regulation may be present at three levels: high or adequate, non-existent, or low. The concept identified as external regulation in Vermunt’s model would be considered a dysregulatory context in the SRL vs. ERL model, because it encourages a lack of internal self-regulation.

(3) *Lack of regulation:* This refers to certain students’ difficulty in regulating their own learning processes. In the SRL vs. ERL model (2017), these students’ level of self-regulation would be categorized as non-regulatory or dysregulatory.

## Learning Approaches (LA) as a Variable in the Teaching and Learning Process

The *SAL model*, Student Approaches to Learning (Marton, 1976; Biggs, 1979; Entwistle et al., 1979; Richardson, 2015; Fryer and Ginns, 2017) established the concept of *learning approaches* (deep vs. surface) as a student variable, with a great amount of empirical evidence (for a review, see Asikainen and Gijbels, 2017). Biggs (1988) defined learning approaches as learning processes that emerge from students’ perceptions of academic tasks, influenced by their personal characteristics. Learning approaches are characterized by the influence of metacognitive processes as a mediating element between the students’ intention or motive and the learning strategy they use in order to study. Biggs indicated two different levels of study in approaches to learning: one is more specific and directed toward a concrete task (a surface approach seen as a process used to pass exams) and the other is more general (a deep approach seen as the motivation to understand). Previous research has associated this variable of learning approaches with learning conceptions (Monroy and González-Geraldo, 2018), with motivational-affective and personal factors (Trigwell et al., 2012; de la Fuente et al., 2013b; Cetin, 2015; Karagiannopoulou et al., 2018), and even with lifelong learning (Barros et al., 2012).

Although fewer in number, other studies have reported its relationship to the teaching process (Vermunt, 1998; Marton et al., 2005; Ruohoniemi et al., 2010). Nonetheless –based on the original conceptualization of this construct– it seems plausible that students’ learning approaches depend on both intra-subject (individual) factors and between-subject (contextual) factors, considering that the nature of the variable is quite subjective, sensitive to diverse influences that stem from the student’s own characteristics as well as from the teacher and from the teaching context (for a review, see Vermunt and Donche, 2017). Consequently, if we assume that the teaching process –teaching approach– affects and has a determining influence on how the student learns –learning approach– (Trigwell et al., 1999), especially in formal contexts, then approaches to learning becomes a variable within the teaching-learning process, not something that pertains only to the student who is learning (Entwistle and Ramsden, 1983; Vermunt and Verloop, 1999; Entwistle et al., 2002, 2003; Vermunt, 2007; Entwistle, 2009, 2018; Parpala et al., 2010; Biggs and Tang, 2011; Baeten et al., 2015). This approach, however, has not been addressed as much as one would expect. In the words of certain authors: “Thus, the effect of the teaching-learning environment is not taken into account so much, despite the largely accepted theoretical assumption in the SAL tradition that students’ approaches to learning are not stable but change as a result of the interaction between the contextual aspects of the learning environment and the characteristics of the learners” (Asikainen and Gijbels, 2017; p. 228). The present study, therefore, adopts this more comprehensive view of student approaches to learning, in the context of teaching and learning processes.

## Academic Achievement and Academic Satisfaction as Variables of the Teaching and Learning Process

### Academic Achievement as a Variable of the Teaching and Learning Process

The classic psychological view of analyzing *academic achievement* has sought to assess the relative weight of students’ individual psychological factors of different types, observing the weight of personal variables, cognitive variables, and motivational-affective variables, as well as others that are psychosocial or contextual (for a meta-analysis review, see Richardson et al., 2012). The educational psychology perspective has led researchers to establish the role of individual psychological factors within a contextualized, specific learning process. There is a great amount of recent research in this regard (Wibrowski et al., 2016; Köller et al., 2019; Nabizadeh et al., 2019), with marked influence from the satisfaction variable, a variable of positive experience and emotionality, in the academic setting (Vanno et al., 2014).

As in the case of learning approaches (LA), there have also been efforts to contextualize achievement within the teaching and learning process (Vermunt, 1998; Biggs and Tang, 2011; Scevak et al., 2015). This approach assumed that academic achievement is determined by variables from both the teaching process and the learning process–taken in combination. In other words, it is not only a matter of the student’s individual variables. Nonetheless, the prevailing view has been to emphasize student variables, assuming that the teaching process has a contextualized role with lesser weight. While this view, which leans heavily toward factors of the learner and is not interrelational with the teaching process, may be adequate in an individual context of learning, it seems unfitted to explaining phenomena in a *formal teaching-learning context*. Hence, while it is true that certain studies have analyzed the role of effective teaching factors in the process of learning and achievement (de la Fuente et al., 2017), a systematic demonstration of the possible combinations of students’ learning characteristics and the teacher’s teaching characteristics is yet to be established. Some prior studies have taken this direction, with encouraging results (de la Fuente et al., 2011). Fewer research studies have documented the role of the *teaching process* as a contributing factor to university students’ academic achievement, despite the fact that most universities assess students’ degree of satisfaction with the teaching process either explicitly or implicitly (Douglas et al., 2015).

*Academic achievement* as a variable has been conceptualized differently. Its classic conceptualization is that of grade point average. Today’s model of achievement, however, is based on the concept of *competence acquisition* (Gagné, 1965) and has prompted consideration of academic achievement as a multidimensional variable that includes acquisitions that are conceptual (facts, concepts and principles), procedural (skills and meta-skills), and *attitudinal* (attitudes, values, and habits) (Roe, 2003; de la Fuente et al., 2004).

### Academic Satisfaction as a Variable of the Teaching and Learning Process

Academic satisfaction with the teaching-learning process has been conceptualized as the emotional or attitudinal element of achievement (Biggs, 2001); it addresses the degree that students’ expectations are met, and how well the process responds to their needs. This variable has been repeatedly considered as an element reflecting the quality of the experience. For example, Bobe and Cooper (2017) defined the category of student *satisfaction with the experience* using five components: teaching quality, learner engagement, learning resources, student support, and skills development. In their sample, Rubin et al. (2018) found that older female students showed the most deep learning, and this effect explained their greater satisfaction with their degree program.

Increasing importance is being given to degree satisfaction (or student satisfaction) for at least two reasons. First, satisfaction predicts student persistence (for a review, see Schertzer and Schertzer, 2004); low satisfaction is an early sign of potential student attrition. Second, satisfaction is a key factor in the rankings of universities, which are commonly used in marketing and funding exercises. Previous findings have shown an association between a deep learning approach and greater satisfaction with teaching and learning environments and methods (Parpala et al., 2010; Gurpinar et al., 2013). Thus, the present study seeks to further our understanding of academic satisfaction, conceptualized as the result of a combination of personal and contextual factors pertaining to the process of teaching and learning.

## Aims and Hypotheses

Based on prior theoretical foundations and previous empirical research, the following *objectives* were identified: (1) to establish whether the university students’ regulation levels (intrapersonal variable) and the regulatory levels of the teaching received (contextual variable), independently, determined their type of learning approach and their academic achievement and satisfaction; (2) to determine whether these levels taken jointly, as described in the combination model proposed by the theory, were associated with the type of learning approach used, academic achievement and satisfaction. Based on these objectives, our *hypotheses* established that: (1) a *graded increase in level of regulation* (internal and external) would give rise to an increase in deep learning approach, and a decrease in surface approach; by contrast, a *graded decrease in level of regulation* (internal and external) would give rise to an increase in surface learning approach and a decrease in deep approach; (2) a *graded increase in level of regulation* (internal and external) would give rise to an proportionate increase in total achievement and in its three subtypes (conceptual, procedural, and attitudinal), and in satisfaction; a *graded decrease in level of regulation* (internal and external) would give a proportionate decrease in total achievement and in its three subtypes (conceptual, procedural, and attitudinal) and satisfaction.

## Materials and Methods

### Participants

A total sample of 1036 undergraduate students from two universities of Spain participated in this research. The sample was composed of students enrolled in degree programs in Psychology and Education (Primary Education); 65.7% were women and 34.3% were men. Their ages ranged from 19 to 25, with a mean age of 21.33 (σ_x_ = 6.9) years.

### Instruments

#### Self-Regulation

This variable was measured using the *Short Self-Regulation Questionnaire* (*SSRQ*) (Brown, 1998; Brown et al., 1999). It has already been validated in Spanish samples (Pichardo et al., 2014; Garzón-Umerenkova et al., 2017). The SSRQ is composed of four factors and 17 items with a consistent confirmatory factor structure (Chi-Square = 250.83, *df* = 112, CFI = 0.95, GFI = 0.94, AGFI = 0.96, RMSEA = 0.059). It has acceptable validity and reliability values as measured by Cronbach’s alpha [total (α = 0.86; Omega = 0.843); goal setting-planning (α = 0.79; Omega = 0.784), perseverance (α = 0.78; Omega = 0.779), decision making (α = 0.72; Omega = 0.718), and learning from mistakes (α = 0.72; Omega = 0.722)], similar to the English version. Sample items include: “I usually keep track of my progress toward my goals,” “When it comes to deciding about a change, I feel overwhelmed by the choice,” and “I learn from my mistakes.”

#### Regulatory Teaching

*The Scales for Assessment of the Teaching-Learning Process, ATLP, student version* (de la Fuente et al., 2012) were used to evaluate the perception of the teaching process in students. The scale entitled *Regulatory Teaching* is Dimension 1 of the confirmatory model. IATLP-D1 comprises 29 items structured along five factors: Specific regulatory teaching, regulatory assessment, preparation for learning, satisfaction with the teaching, and general regulatory teaching. The scale showed a factor structure with adequate fit indices (Chi-Square = 590.626; *df* = 48, *p* < 0.001, CF1 = 0.958, TLI = 0.959, NFI = 0.950, NNFI = 0.967; RMSEA = 0.068) and adequate internal consistency [IATLP 1 Scale (α = 0.830; Omega = 0.821), and the subscales: Specific regulatory teaching (α = 0.897; Omega = 0.852); regulatory assessment (α = 0.883; Omega = 0.876); preparation for learning (α = 0.849; Omega = 0.835); satisfaction with the teaching, (α = 0.883; Omega = 0.861), and general regulatory teaching, (α = 0.883; Omega = 0.858)]. Sample items include: “While we are learning the teacher help us to make clear realistic learning goals,” “The teacher explains the objetives of activities we are going to carry out,” or “The teacher make the class enjoyable.”

#### Learning Approaches

This was measured with the Revised Two-Factor Study Process Questionnaire, R-SPQ-2F (Biggs et al., 2001), in its Spanish validated version (Justicia et al., 2008). It contains 20 items on four subscales (deep motive, deep strategy; surface motive, surface strategy), measuring two dimensions: deep and surface learning approaches, respectively. Students respond to these items on a 5-point Likert-type scale ranging from 1 (rarely true of me) to 5 (always true of me). In the present study Cronbach’s alpha reliability coefficients were acceptable: Deep (α = 0.793; Omega = 0.782); Surface (α = 0.751; Omega = 0.721). Sample items include: “I find that at times studying gives me a feeling of deep personal satisfaction,” “My aim is to pass the course while doing as little work as possible,” “I find that studying academic topics can at times be as exciting as a good novel or movie.”

#### Academic Achievement

Assessment of achievement was based on the academic-professional competency model (Roe, 2003). Total achievement was measured as the final grade given to the student for the subject, on a scale of 1 to 10. The 10 points are a compendium of results obtained on the three levels of subcompetencies: (1) *Conceptual* scores: these include all scores obtained on exams covering the conceptual content of the subject (4 points); (2) *Procedural* scores: assessed from the student’s practical work involving procedural content and skills (4 points); (3) *Attitudinal* scores: scores given for class participation, and for doing optional activities to reach a better understanding of the material (2 points). In the latter case, there were 10 class activities that were turned in at the end of class; the mean of the 10 scores obtained was converted proportionately to a score on the 0−2 point range. Since the three subcompetencies were measured on different ranges (0−4 points, 0−2 points), their scores were converted to an equivalent scale from 1 to 10 in order to perform the different analyses and compare the results.

#### Satisfaction With Teaching and Learning

*The Scales for Interactive Assessment of the Teaching-Learning Process*, IATLP, student version (de la Fuente et al., 2012) were used to evaluate students’ perception of the teaching process. The scale entitled *Satisfaction of teaching and Learning* is Dimension 3 of the confirmatory model (IATLP-D3). This sub-scale comprises 10 items structured along two factors. The scale was validated in university students and showed a factor structure with adequate fit indices (Chi-Square = 590.626; *df* = 48, *p* < 0.001, CF1 = 0.938, TLI = 0.939, NFI = 0.950, NNFI = 0.967; RMSEA = 0.058) and adequate internal consistency [IATLP D3 (α = 0.85; Omega = 0.831); *Satisfaction with learning process* (α = 0.86; Omega = 0.831); and *Satisfaction with teaching process* (α = 0.87; Omega = 0.861)]. Sample items include: “I am satisfied with the way my teacher has carried out the teaching” and “I am satisfied with the way I have learned.”

### Procedure

Students voluntarily completed the scales using an online platform (de la Fuente et al., 2015a). A total of fifteen specific teaching-learning processes were evaluated, each pertaining to a specific university subject that was taught within a 2-year academic period. *Presage* variables (Self-regulation, SR) were evaluated in September-October of 2017 and of 2018, *Process* variable (learning approaches, LA) in February-March of 2017 and of 2018, and *Product* variables (regulatory teaching, satisfaction with teaching and learning process, and academic achievement) in May-June of 2017 and of 2018. Achievement was reported by the teacher, based on the academic grades that students obtained at the end of the school year. In all cases, scores had been assigned for the three types of subcompetencies (conceptual, procedural, and attitudinal). Cases were eliminated if any of these scores were lacking.

At each university, teachers were invited to participate in the research project; once they agreed, they in turn invited the participation of their students. Each group of students evaluated only one teacher and the teaching-learning process of one full-year academic subject. The teachers and students received a certificate acknowledging their hours of participation in the project. In no case was any academic credit given for participation. The procedure was approved by the respective Ethics Committees at each university, in the context of the two R & D Projects (see Funding).

### Data Analysis

#### Design

An *ex post* facto design was used. There was no intervention of any kind in the teaching-learning processes assessed. Only pre-existing variables were evaluated.

#### Previous Analysis

Preliminary analyzes were carried out to detect different problems in the sample data. About the potential outliers in the data, univariate outliers were identified by checking standardized scores on any variables which were outside the absolute value of 3.29 (Tabachnick and Fidell, 2013). Complementary, to detect multivariate outliers, Mahalanobis distance (MD) for the predictor variables were used, which is the distance of a data point from the centroid shaped by the cloud of the majority of data points (Mahalanobis, 1930). In this process, 21 cases were eliminated. Regarding the reliability of the scales used, the omegaH index has been recalculated; for those multidimensional variables, it is essential to provide model-based reliability (for both general factor and specific sub-factors) rather than simply reporting Alpha (Reise et al., 2012).

#### Operationalization of Self- vs. External- Regulation

Using cluster analysis, continuous independent variables were converted into discrete, dependent variables, producing three levels (low-medium-high) for self-regulation and regulatory teaching, respectively. The centroids of low, medium, and high scoring groups were calculated in each variable. Next, we determined the cutoff points between scores. In this way, we established the score ranges for low (L), medium (M), and high (H) (see Table 1, on the left, in boldface).

#### Inferential Analyses

Different ANOVAs and MANOVAs were carried out, taking high/medium/low levels of SR and RT as independent variables. First, we performed 3 × 1 (simple) and 3 × 3 (cross) analyses.

#### A Heuristic of Regulation Combinations for the Teaching and Learning Process

Finally, the MANOVA (5 × 1) showed statistically significant differences in the levels of variables SR and RT among the five groups, showing them to be adequately configured. This procedure was similar to that used in other previous reports (de la Fuente et al., 2019b, p. 12; de la Fuente et al., 2020a, p. 5). The multivariate analyses (MANOVAs) showed a statistically significant main effect of the five combination types on low-medium-high levels of SR and of RT (see Table 1):

*Combination* 1 presented a statistically significant low level in SR and low level in RT (*1 and 1 levels*). The effects are a high level of surface approach, low level of deep approach, low level of achievement, and low level of satisfaction.

*Combination* 2 had a statistically significant low level in SR and medium level in RT, or viceversa (*1 and 2, or 2 and 1 levels*). The effects are a medium-high level of surface approach, medium-low level of deep approach, medium-low level of achievement, and medium-low level of satisfaction.

*Combination* 3 presented a statistically significant medium SR level (*2*) and medium RT level (*2 and 2 levels*). The effects are a medium level of surface approach, medium level of deep approach, medium level of achievement, and medium level of satisfaction.

*Combination* 4 had a statistically significant medium level in *SR* and high level in *RT*, or viceversa (*2 and 3*, or *3 and 2 levels*). The effects are a medium-high level of deep approach, medium-low level of surface approach, medium-high level of achievement, and medium-high level of satisfaction.

*Combination* 5 presented statistically significant high SR and high RT (*3 and 3 levels*). The *average regulation level is 3.0, and its regulation rank is 5*. The effects are a high level of deep approach, low level of surface approach, high level of achievement, and high level of satisfaction.

The proposed five-combination heuristic enables us to analyze all the most common combinations found in the interactive regulation of teaching-learning processes. A *regulation average* is obtained from the student-teaching interaction by calculating the mean of the student’s regulation level and the regulation level of the teaching process. For example, if the student has a low level of regulation (1 point), and the teaching offers a medium level of regulation (2 points), the resulting regulation average will be 1.5 points (2 + 1 = 3/2 = 1.5 point average). Inversely, a student with medium regulation (2 points) and a teaching process low in regulation (1 point) would result in the same regulation average (2 + 1 = 3/2 = 1.5 average points). In another case, if a student has a high level of regulation (3 points) and interacts with teaching that is low in regulation (1 point), the regulation average will be 2 points (3 + 1 = 4/2 = 2 points). The student-teaching interaction increases from the least favorable to the most favorable: the minimum combination of low student regulation (1 point) with teaching low in regulation (1 point), to a maximum combination of high student regulation (3 points) with highly regulatory teaching (3 points). The heuristic then orders all the possible combinations according to their regulation average, assigning to them a *regulation rank* (regulation average of 1 = rank 1; regulation average of 1.5 = rank 2; regulation average of 2 = rank 3; regulation average of 2.5 = rank 4; regulation average of 3 = rank 5).

## Results

### Interdependent Effects of Levels of Personal Self-Regulation (SR) and Levels of Regulatory Teaching (RT) on Learning Approaches, Academic Achievement, and Satisfaction

#### Effects on Dimensions and Factors of Learning Approaches (LA)

There was a statistically significant main effect of *Self-Regulation* (SR) on the two dimensions of learning approach (LA): Deep Approach and Surface Approach. The effect of SR (low-medium-high levels) was statistically significant for both deep approach and surface approach. A higher level of SR determined a higher level of deep approach and a lower level of surface approach. Complementarily, a lower level of SR determined a lower level of deep approach and a higher level of surface approach. See Table 2 (first part of the table, on the left).

**TABLE 2:** Simple interdependent relations of low-medium-high levels of *Self-Regulation* (SR) and of *Regulatory Teaching* (RT), as independent variables, on *Learning Approaches* (*n* = 1209).

*DVs*	*VI Self-Regulation level* (*SR*)				*F*(*Pillai’s*)	*Post hoc*	*VI. Regulatory Teaching level* (*RT*)				*F*(*Pillai’s*)	*Post hoc*
	1. *Low*	*2. Medium*	*3. High*	*Mean*			*1*. *Low*	*2. Medium*	*3. High*			
	(*n* = 321)	(*n* = 553)	(*n* = 335)	(*n* = 1209)			(*n* = 198)	(*n* = 495)	(*n* = 343)	(*n* = 1036)		
*LA Dimensions*					*F*(4,1926) = 31.685**, *n^2^* = 0.089,	*p* = 1.0					*F* (4, 1924) = 8.820**, *n*^2^ = 0.030,	*p* = 1.0
DA	2.71 (0.54)	2.94 (0.56)	3.28 (0.58)	2.97 (0.60)	*F* (2, 963) = 35.611**, *n*^2^ = 0.123,	1 < 2 < 3	2.80 (0.58)	2.88 (0.54)	3.16 (0.64)	2.94 (0.60)	*F* (2, 963) = 16.381**, *n*^2^ = 0.050,	1,2 < 3
SA	2.44 (0.58)	2.16 (0.54)	1.89 (0.54)	2.16 (0.59)	*F* (2, 963) = 49.828**, *n^2^* = 0.094,	1 > 2 > 3	2.26 (0.62)	2.20 (0.55)	2.06 (0.61)	2.16 (0.59)	*F* (2, 963) = 2.735**, *n^2^* = 0.006,	1, 2 > 3
*LA Factors*					*F* (8,1922) = 16.594**, *n^2^* = 0.065, *p* = 1.0	1 > 2 > 3					*F* (8,1922) = 4,704**, *n^2^* = 0.032,	*p* = 1.0
DM	2.84 (0.60)	3.12 (0.60)	3.43 (0.63)	3.13 (0.65)	*F* (2, 963) = 30.524**, *n^2^* = 0.060,	1 < 2 < 3	2.94 (0.65)	3.06 (0.61)	3.23 (0.65)	3.12 (0.65)	*F* (2, 963) = 14.957**, *n*^2^ = 0.030,	1 < 2 < 3
DS	2.59 (0.63)	2.75 (0.64)	3.13 (0.65)	2.81 (0.67)	*F* (2, 963) = 27.533**, *n^2^* = 0.054,	1 < 2 < 3	2.66 (0.67)	2.71 (0.62)	3.00 (0.72)	2.80 (0.68)	*F* (2, 963) = 11.634**, *n*^2^ = 0.024	1,2 < 3
SM	2.09 (0.64)	1.82 (0.56)	1.58 (0.53)	1.83 (0.60)	*F* (2, 963) = 39.925**, *n^2^* = 0.077,	1 > 2 > 3	1.93 (0.66)	1.85 (0.67)	1.72 (0.71)	1.82 (0.80)	*F* (2, 963) = 3,345**, *n^2^* = 0.024	1,2 > 3
SS	2.80 (0.48)	2.49 (0.64)	2.20 (0.65)	2.49 (0.48)	*F* (2, 963) = 41.188**, *n^2^* = 0.080,	1 > 2 > 3	2.59 (0.72)	2.54 (0.74)	2.40 (0.70)	2.50 (0.66)	*F* (2, 963) = 1.514**, *n^2^* = 0.003	1, 2 > 3

***DVs***	***Self-Regulation***					***Regulatory Teaching***					***F* (*Pillai’s Trace*)**	***Post hoc***
	**1. Low**	**2. Medium**	**3. High**	**Average**			**1. Low**	**2. Medium**	**3. High**	**Average**		
	** (*n* = 193)**	** (*n* = 340)**	** (*n* = 257)**	** (*n* = 790)**			** (*n* = 150)**	** (*n* = 321)**	** (*n* = 216)**	** (*n* = 687)**		

**Achievement**												
Total	2.91 (1.2)	3.16 (0.1.2)	3.60 (1.3)	3.24 (1.2)	*F* (2,632) = 7.024**, *n*^2^ = 0.022, *p* = 0.98;	1 < 2 < 3	2.55 (1.2)	3.20 (1.2)	3.70 (1.2)	3.22 (1.3)	*F* (2,637) = 22.880**, *n*^2^ = 0.067, pow = 1.0;	1 < 2 < 3
					*F* (6,1262) = 4.763**, *n*^2^ = 0.034, pow = 1.0						*F* (6,1262) = 4.470**, *n*^2^ = 0.021, power = 0.98	
Conceptual	2.88 (0.73)	3.06 (0.70)	3.32 (0.69)	3.10 (0.72)	*F* (2, 787) = 22.101**, *n*^2^ = 0.053,	1 < 2 < 3	2.86 (0.76)	3.08 (0.65)	3.31 (0.62)	3.10 (0.74)	*F* (2,632) = 8.498**, *n*^2^ = 0.026,	1 < 2 < 3
Procedural	2.87 (0.77)	3.10 (0.71)	3.35 (0.69)	3.12 (0.73)	*F* (2, 787) = 24.612**, *n*^2^ = 0.059,	1 < 2 < 3	2.83 (0.82)	3.10 (0.69)	3.33 (0.64)	3.11 (0.73)	*F* (2,632) = 12.784**, *n*^2^ = 0.039,	1 < 2 < 3
Attitudinal	1.82 (0.34)	1.87 (0.39)	1.91 (0.53)	1.87 (0.33)	*F* (2, 787) = 3.357**, *n*^2^ = 0.035,	1 < 2 < 3	1.79 (0.41)	1.87 (0.34)	1.92 (0.27)	1.87 (0.34)	*F* (2, 632) = 3.209**, *n*^2^ = 0.010,	1 < 2 < 3
*Satisfaction*	3.48 (0.66)	3.80 (0.57)	4.17 (0.54)	3.82 (0.64)	*F* (2, 1129) = 47.441**, *n*^2^ = 0.154, pow = 1.0;	1 < 2 < 3	3.32 (0.68)	3.71 (0.53)	4.25 (0.46)	3.82 (0.62)	*F* (2,942) = 142.903**, *n*^2^ = 0.233, pow = 1.0;	1 < 2 < 3

** p < 0.001. DA, deep approach; SA, surface approach; DM, deep motivation; SM, surface motivation; DS, deep strategy; SS, surface strategy.

Complementarily, there was a statistically significant main effect of SR (low-medium levels) on learning approach factors. The partial effect of SR (low-medium-high levels) was statistically significant for the factors of deep motivation, deep strategy, surface motivation, and surface strategy. A higher level of SR determined a higher level of the factors deep motivation and deep strategy, and a lower level of surface motivation and surface strategy. A lower level of SR determined the opposite case, that is, a lower level of deep motivation and deep strategy, and a higher level of surface motivation and surface strategy. See Table 2 (first part of the table, on the left) and Table 3.

**TABLE 3:** Combined effects (3 × 3) between levels of Self-Regulation (SR) and levels of Regulatory *Teaching* (*RT*) on *Learning Approches* (*n* = 972).

*SR*	*Low*	(*n* = *257*)		*Medium*	(*n* = *451*)		*High*	(*n* = *264*)		*Total*		*F* (*Pillais*)	*Post hoc*
*RT*	*Low*	*Med*	*High*	*Low*	*Med*	*High*	*Low*	*Med*	*High*				
*n* =	62	140	55	84	227	240	32	103	129	(*n* = 972)			
*LA Dimensions*											GrupSR	*F* (4,1926) = 31,685**, *r*^2^ = 0.062	
											GrupRT	*F* (4,1926) = 8,820**, *r*^2^ = 0.062	
DA	2.56 (0.53)	2.67 (0.48)	2.94 (0.59)	2.88 (0.57)	2.90 (0.53)	3.06 (0.63)	3.10 (0.53)	3.13 (0.54)	3.36 (0.61)	2.96 (0.60)	GrupSR	*F* (2,963) = 35,611**, *r*^2^ = 0.069	3 > 2 > 1**
											GrupRT	*F* (2,963) = 16,381**, *r*^2^ = 0.033	3,2 > 1**
SA	2.59 (0.58)	2.45 (0.55)	2.34 (0.58)	2.18 (0.60)	2.16 (0.62)	2.08 (0.58)	1.94 (0.55)	1.90 (0.50)	1.89 (0.60)	1.91 (0.55)	GrupSR	*F* (2,963) = 49,828**, *r*^2^ = 0.094	1 > 2 > 3**
											Grup RT	*F* (2,963) = 2,735*, *r*^2^ = 0.003	1 > 2,3**
*LA Factors*											GrupSR	*F* (8,1992) = 16,594**, *r*^2^ = 0.065	
											Grup RT	*F* (8,1992) = 4,704**, *r*^2^ = 0.019	
DM	2.68 (0.63)	2.80 (0.58)	3.09 (0.59)	3.05 (0.65)	3.10 (0.57)	3.24 (0.65)	3.21 (0.60)	3.28 (0.61)	3.51 (0.64)	3.12 (0.65)	GrupSR	*F* (2,963) = 30,542**, *r*^2^ = 0.060	3 > 2 > 1**
											Grup RT	*F* (2,963) = 14,957**, *r*^2^ = 0.030	3 > 2,1**
DS	2.44 (0.59)	2.54 (0.55)	2.79 (0.72)	2.72 (0.64)	2.69 (0.62)	2.89 (0.70)	2.98 (0.67)	3.00 (0.58)	3.20 (0.71)	2.80 (0.67)	GrupSR	*F* (2,963) = 27,533**, *r*^2^ = 0.077	1 > 2 > 3**
											Grup RT	*F* (2,963) = 11,634**, *r*^2^ = 0.024	3 > 2,1**
SM	2.26 (0.67)	2.10 (0.60)	1.94 (0.62)	1.86 (0.65)	1.82 (0.52)	1.74 (0.60)	1.60 (0.49)	1.58 (0.59)	1.58 (0.51)	1.83 (0.61)	GrupSR	*F* (2,963) = 39,925**, *r*^2^ = 0.062	1,2 > 3**
											Grup RT	*F* (2,963) = 3,445*, *r*^2^ = 0.007	
SS	2.92 (0.69)	2.80 (0.62)	2.74 (0.64)	2.49 (0.69)	2.54 (0.60)	2.43 (0.67)	2.21 (0.68)	2.50 (0.66)	2.20 (0.70)	2.51 (0.68)	GrupSR	*F* (2,963) = 41,778**, *r*^2^ = 0.080	1 > 2 > 3**
											GrupRT	*F* (2,963) = 1,514^ns^, *r*^2^ = 0.080	
*Academic Achievement Total*	2.38 (1.1)	2.91 (1.7)	3.37 (1.3)	2.147 (1.2)	3.20 (1.2)	3.59 (1.1)	3.04 (1.4)	3.33 (1.0)	3.91 (1.9)	3.21 (1.3)	GrupSR	*F* (2,637) = 7,0345**, *r*^2^ = 0.034; pow = 0.98	3,2 > 1**
											GrupRT	*F* (2,637) = 22,880**, *r*^2^ = 0.067; pow = 1,0	3 > 2 > 1**
											GrupSR	*F* (6,1646) = 4,763**, *r*^2^ = 0.022; pow = 0.91	
											GrupRT	*F* (6,1262) = 4,470**, *r*^2^ = 0.021; pow = 0.986	
*Conceptual* (*4p*)	2.72 (0.70)	2.91 (0.71)	2.97 (0.79)	2.86 (0.79)	3.06 (0.76)	3.26 (0.60)	3.08 (0.90)	3.24 (0.54)	3.46 (0.63)	3.10 (0.73)	GrupSR	*F* (2,632) = 11,663**, *r*^2^ = 0.036; pow = 0.994	3 > 2 > 1*
											GrupRT	*F* (2,632) = 8,848**, *r*^2^ = 0.026; pow = 0.966	3,2 > 1**
*Procedural* (*4p*)	2.54 (0.75)	2.91 (0.78)	3.13 (0.86)	2.95 (0.82)	3.10 (0.63)	3.26 (0.58)	3.08 (0.90)	3.26 (0.69)	3.48 (0.60)	3.12 (0.73)	GrupSR	*F* (2,632) = 12,238**, *r*^2^ = 0.037; pow = 0.996	3 > 2 > 1**
											GrupRT	*F* (2,632) = 12,748**, *r*^2^ = 0.039; pow = 0.997	3,2 > 1**
*Attitudinal* (*2p*)	1.78 (0.47)	1.82 (0.38)	1.81 (0.40)	1.75 (0.43)	1.87 (0.33)	1.94 (0.24)	1.84 (0.37)	1.91 (0.29)	1.93 (0.29)	1.86 (0.34)	GrupSR	*F* (2,632) = 2,528*, *r*^2^ = 0.008; pow = 0.506	3 > 2 > 1*
											GrupRT	*F* (2,632) = 3,209*, *r*^2^ = 0.010; pow = 0.613	3,2 > 1**
*Satisfaction*	3.03 (0.61)	3.52 (0.51)	3.96 (0.46)	3.38 (0.59)	3.73 (0.49)	4.19 (0.43)	3.63 (0.64)	3.95 (0.43)	4.40 (0.40)	3.82 (0.63)	GrupSR	*F* (2,972) = 53,406**, *r*^2^ = 0.099; pow = 1.0	3 > 2 > 1**
											GrupRT	*F* (2,972) = 222,876**, *r*^2^ = 0.350; pow = 1.0	3 > 2 > 1**

GrupSR, Effect of IV level in Self-Regulation; GrupRT, Effect of IV level in Regulatory Teaching; *p < 0.05, **p < 0.001, ns, non-significant statistical effect. DA, deep approach; SA, surface approach; DM, deep motivation; SM, surface motivation; DS, deep strategy; SS, surface strategy.

There was a statistically significant main effect of *Regulatory Teaching* (RT) (low-medium-levels) on Learning Approach dimensions. The partial effect of RT (low-medium-high levels) was statistically significant for both deep approach (DA) and surface approach (SA). Thus, a higher level in regulatory teaching determined a higher level in DA and a lower level in SA; by contrast, a lower level in RT determined a higher level in SA and lower level in DA. Complementarily, there was a statistically significant main effect of RT (low-medium levels) on learning approach factors. The partial effect of RT (low-medium-high levels) was statistically significant for the factors of deep motivation (DM), deep strategy (DS), surface motivation (SM), and surface strategy (SS). Accordingly, a higher level of RT determined a higher level of DM and DS, and lower levels of SM and SS. By contrast, lower levels of RT determined higher levels of SM and SS and lower levels of DM and DS. See Table 2 (first part of the table, on the right) and Table 3.

#### Effects on Academic Achievement (ACH) and Satisfaction (SAT)

**FIGURE 1:**
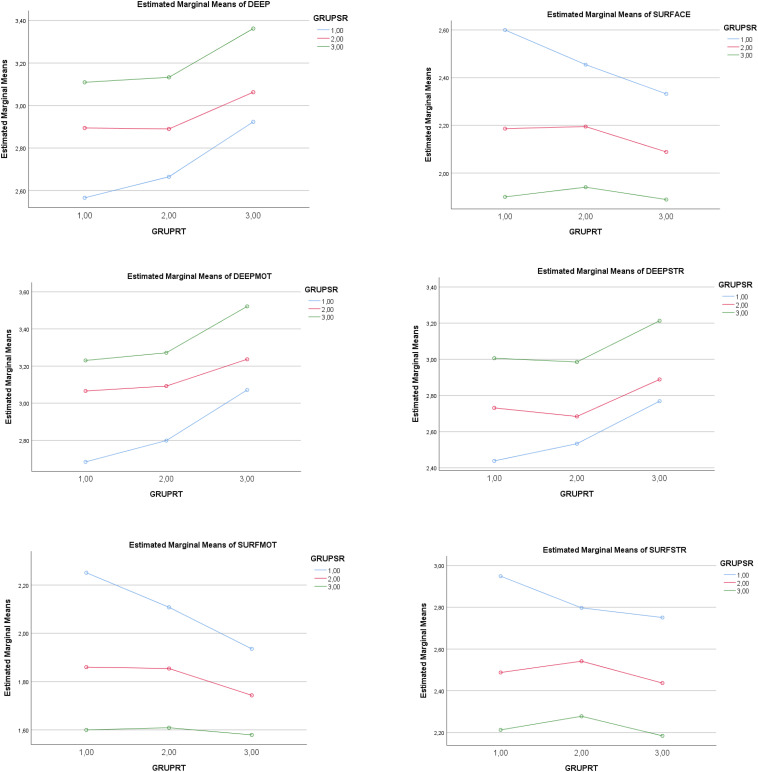
Graphic representation of the effect of levels in the IV *Self-Regulation* (GRUPSR: Low = 1; Medium = 2; High = 3) and level in the IV *Regulatory Teaching* (GRUPRT: Low = 1; Medium = 2; High = 3) on *Learning Approaches* (LA). DEEP, Deep approach; SURFACE, Surface approach; DEEPMOT, Deep motivation; DEEPSTRAT, Deep strategies; SURFMOT, Surface motivation; SURFSTRAT, Surface strategies.

**FIGURE 2:**
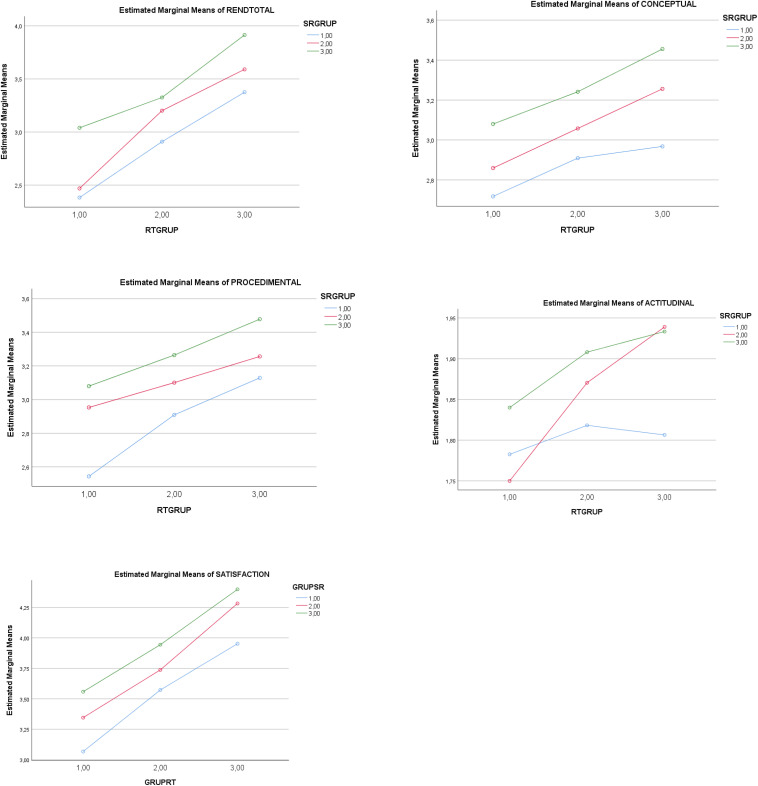
Graphic representation of the effect of low(1)-medium(2)-high(3) levels in the IV *self-regulation* (GRUPSRQ) and low(1)-medium (2)-high (3) levels in the IV *regulatory teaching* (GRUPER) on *academic achievement* (*conceptual*, *procedural*, *and attitudinal*) and *satisfaction with the teaching and learning process.*

There was a statistically significant main effect of SR (low-medium levels) on total academic achievement (ACH). A higher level of SR determined a higher total achievement score, and a lower level did the opposite. Complementarily, there was a statistically significant main effect of SR (low-medium levels) on the ACH factors. The partial effect of SR (low-medium-high levels) was statistically significant for the factors of *conceptual achievement, procedural achievement*, and *attitudinal achievement*. In other words, a higher score in SR determined a higher level in the three types of achievement. Complementarily, there was a statistically significant effect of SR (*low-medium levels*) on *academic satisfaction* (*SAT*). In similar fashion, a higher level of SR determined a higher level of SAT, and a lower level did the opposite. See Table 2 (second part, on the left), Table 3, and Figures 1, 2.

There was a statistically significant main effect of RT (low-medium levels) on total ACH. A higher level in RT determined a higher level in ACH. Complementarily, there was a statistically significant main effect of RT (low-medium levels) on the ACH factors. The partial effect of RT (low-medium-high levels) was statistically significant for the factors of *conceptual achievement*, *procedural achievement*, and *attitudinal achievement*. A high level of RT, therefore, was a determinant of higher levels in all three types of achievement. Complementarily, a statistically significant effect of RT (low-medium-levels) was noted in *academic satisfaction.* Thus, a higher level of RT determined a higher level of SAT. See Table 2 (second part), Table 3, and Figures 1, 2.

It is important to emphasize that interaction effects between SR and RT were not produced, but main effects from each variable independently, making an additive effect. The following section documents this summative effect using the combination heuristic.

### Combination Heuristic of SR vs. ER: Understanding Its Effect on Learning Approaches, Academic Achievement, and Satisfaction

#### Effects of the Combination Heuristic on Learning Approaches

A statistically significant main effect of the *five combinations of SR and RT* was observed in learning approaches (LA). In the dimensions of deep approach (DA) [5 > 4 > 3 > 2,1] and surface approach (SA) [1,2 > 3 > 4,5], a significant statistical effect also appeared, but in opposing directions. These results show that higher levels of the heuristic combination determined higher levels of DA and lower levels of SA; by contrast, lower levels of the combination heuristic determined lower levels of DA and higher levels of SA. See Figure 1 and Table 4.

**TABLE 4:** Effects of the Five Types of Combinations on *Learning Approaches* (LA) and *Academic Achievement and Satisfaction*.

DVs	Combination Types in Groups (IVs)						
	1	2	3	4	5	*F* (*Pillai’s Trace*)	*Post hoc*
	(*n* = 63)	(*n* = 236)	(*n* = 338)	(*n* = 253)	(*n* = 140)	(*n* = 972)	
*Configuration Group*						*F* (8,2050) = 187.65**, *n^2^* = 0.423	
*Self-Regulation*	2.65 (0.37)	3.02 (0.42)	3.41 (0.44)	3.80 (0.39)	4.23 (0.29)	*F* (4,1025) = 302.61**, *n^2^* = 0.541	all *p* < 0.001
*Regulatory Teaching*	2.73 (0.32)	3.24 (0.50)	3.63 (0.48)	4.03 (0.44)	4.39 (0.29)	*F* (4,1025) = 252.64**, *n^2^* = 0.496	all *p* < 0.001
*LA Dimensions*						*F* (2,1934) = 22.083,**, *n^2^* = 0.084, pow = 1.0	
DA	56 (0.53)	2.75 (0.52)	2.92 (0.54)	3.09 (0.59)	3.36 (0.61)	*F* (4,967) = 35.116**, *n^2^* = 0.127	5 > 4 > 3 > 2,1**
SA	59 (0.58)	2.35 (0.59)	2.18 (0.53)	2.02 (0.55)	1.89 (0.60)	*F* (4,967) = 26.109**, *n^2^* = 0.097	1,2 > 3 > 4,5**
*LA Factors*						*F* (16,38682) = 11,230**, *n^2^* = 0.044, pow = 1.0	
DM	2.68 (0.63)	2.89 (0.62)	3.11 (0.58)	3.26 (0.63)	3.51 (0.64)	*F* (4,967) = 31.129**, *n^2^* = 0.114	5,4 > 3,2 > 1**
DS	2.44 (0.59)	2.61 (0.59)	2.74 (0.75)	2.93 (0.65)	3.21 (0.71)	*F* (4,967) = 25.681**, *n^2^* = 0.096	5,4 > 3,2 > 1**
SM	2.26 (0.67)	2.01 (0.63)	1.82 (0.64)	1.68 (0.56)	1.58 (0.55)	*F* (4,967) = 23.478**, *n^2^* = 0.089	1,2 > 3 > 4,5**
SS	2.92 (0.69)	2.68 (0.66)	2.54 (0.63)	2.36 (0.66)	2.20 (0.70)	*F* (4,967) = 20.190**, *n^2^* = 0.077	1,2 > 3 > 4,5**

	***Combination Types in Groups* (*IVs*)**						
**DVs**	**1**	**2**	**3**	**4**	**5**	***F* (*Pillai’s Trace*)**	***Post hoc***
	** (*n* = 47)**	** (*n* = 141)**	** (*n* = 196)**	** (*n* = 169)**	** (*n* = 93)**	** (*n* = 646)**	

*Total Achievement*	2.38 (1.11)	2.71 (1.12)	3.21 (1.2)	3.46 (1.2)	3.91 (1.1)	*F* (4,641) = 20,451**, *n^2^* = 0.113, pow = 1.0	5 > 4,3 > 2,1**
Conceptual	2.72 (0.72)	2.89 (0.74)	3.05 (0.72)	3.25 (0.57)	3.46 (0.63)	*F* (4,636) = 15.592**, *n^2^* = 0.089	5,4 > 3,2 > 1**
Procedural	2.54 (0.71)	2.93 (0.79)	3.10 (0.72)	3.26 (0.60)	3.48 (0.70)	*F* (4,636) = 18.145**, *n^2^* = 0.102	5 > 4 > 3,2 > 1**
Attitudinal	1.78 (0.41)	1.79 (0.41)	1.86 (0.35)	1.92 (0.16)	1.93 (0.25)	*F* (4,636) = 4.723**, *n^2^* = 0.029	5,4 > 3 > 2,1**
*Satisfaction*	3.03 (0.61)	3.47 (0.58)	3.76 (0.51)	4.09 (0.44)	4.44 (0.40)	*F* (4,946) = 128.597**, *n^2^* = 0.352, pow = 1.0	5 > 4 > 3 > 2 > 1**

Type 1 (low self-regulation, and low regulatory teaching); Type 2 (low self-regulation and high regulatory teaching); Type 3 (medium self-regulation and medium regulatory teaching); Type 4 (high self-regulation and low regulatory teaching); Type 5 (high self-regulation and high regulatory teaching). DA, deep approach; SA, surface approach; DM, deep motivation; DS, deep strategy; SM, surface motivation; SS, surface strategy. **p < 0.001.

**FIGURE 3:**
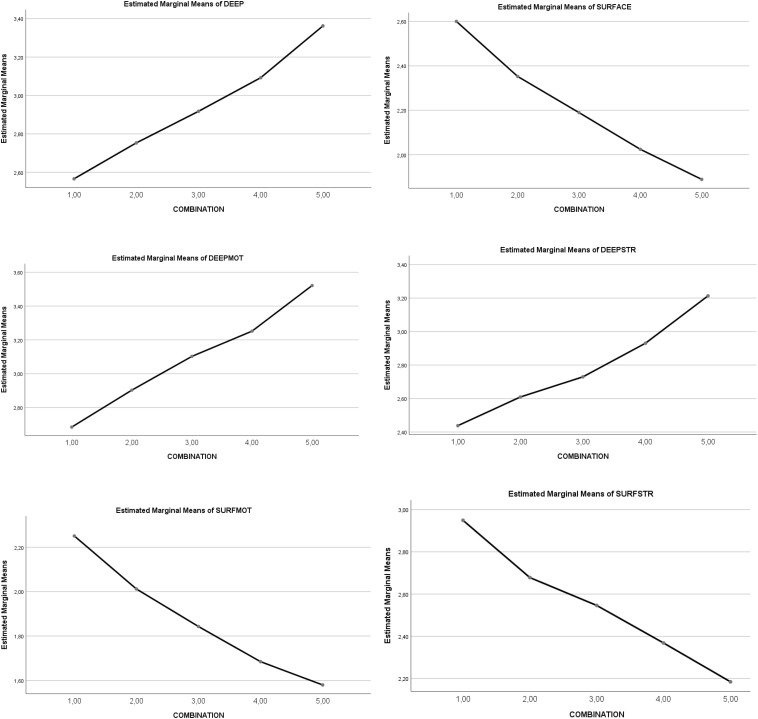
Graphical representation of the effects of the combination types (heuristic levels 1–5) on *learning approaches.*

The statistically significant partial effect was maintained for each factor: deep motivation (DM) [5,4 > 3,2 > 1] and deep strategies (DS) [5,4 > 3,2 > 1], surface motivation (SM) [1,2 > 3 > 4,5], and surface strategies (SS) [1,2 > 3 > 4,5]. High levels of the heuristic determined high levels in DM and DS, as well as low levels in SM and SS; however, low levels of the heuristic determined low levels in DM and DS, as well as high levels in SM and SS. See Figure 3 and Table 4. A graphic representation of the differential progressive effect of the combinations of SR and RT levels is shown in Figure 3.

### Effects of the Combination Heuristic on Academic Achievement and Satisfaction

**FIGURE 4:**
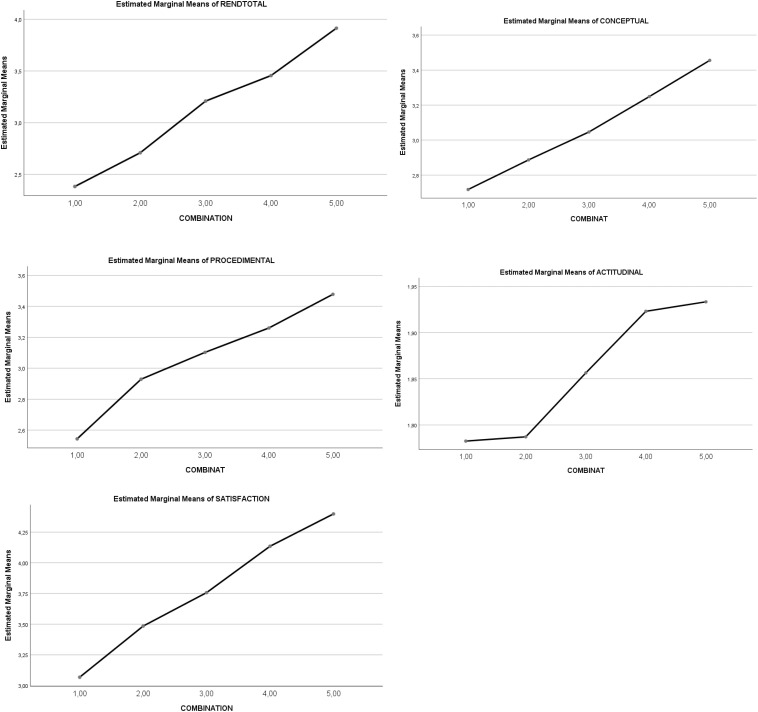
Graphical representation of the effects of the combination types (heuristic levels 1–5) on *academic achievement* and *satisfaction.*

A statistically significant main effect of the five *combinations of IVs SR and RT* was noted on *total achievement* [5 > 4, 3 > 2,1]. The statistically significant partial effect was maintained for each factor: *conceptual achievement* [5,4 > 3,2 > 1], *procedural achievement* [5 > 4 > 3,2 > 1], and *attitudinal achievement* [5,4 > 3 > 2,1]. Complementarily, a statistically significant main effect of the *five combinations of the IVs SR and RT* was noted on *satisfaction* [5 > 4 > 3 > 2 > 1]. See Figure 4 and Table 4. A graphic representation of the differential progressive effect of the combinations of SR and RT levels is shown in Figure 4.

## Discussion

### Implications for the Knowledge of This Research Topic

#### Effects on Learning Approaches

*Self-Regulated Learning vs. ERL Theory* (de la Fuente, 2017) predicted that university students’ learning approaches, academic achievement and satisfaction could be determined, jointly, by the students’ degree of *self-regulation* (SR) and by the level of contextual or *external regulation* (RT). Furthermore, this type of interaction could be understood by the combination of *low-medium-high levels* of the two factors (SR and RT), as supported by prior evidence in this direction (de la Fuente et al., 2017).

With respect to the *first hypothesis*, the evidence found upholds the theory that a surface vs. deep learning approach is a student-dependent variable, depending on the student’s pre-existing level of self-regulation (Heikkilä and Lonka, 2006; de la Fuente et al., 2008). Interestingly, however, other novel data presented here have shown that a high level of SR more strongly determines the level of deep motivation, but not so much the level of deep strategies, and viceversa, a *low level of SR* determines a greater number of surface strategies. These differentiating details had not been clearly established to date, and have implications for assessment and improved psychoeducational intervention – to be further discussed below. This result is consistent with the evidence showing that excellent students have a higher level of deep approach in comparison to average students (Gargallo et al., 2015).

In complementary fashion, a *high level of RT* (regulatory or effective teaching) has been shown to promote a greater degree of the deep learning approach; a *low level of RT* promotes a surface learning approach. Moreover, a differentiating effect was found, where *highly regulatory teaching* was clearly seen to have a greater effect on deep motivation than on deep strategy, while *low regulatory teaching* has more effect on surface strategy than on surface motivation. In other words, good (regulatory) teaching encourages motivation more than high-level cognitive processes, while less regulatory teaching (non-regulatory or dysregulatory) seems to lead to poorer cognitive processes, and learning processes *per se*, more than it affects surface motivation. This effect is novel, and seems to allude to a differential effect of teaching on cognitive and motivational processes, which must be analyzed in greater depth.

From our point of view, however, the most interesting effect found here is the effect produced by the *combination* of student characteristics and characteristics of the teaching process, in determining university students’ *learning approach.* All the cross analyses and especially the heuristic-based analyses themselves (graded combinations 1−5), have consistently supported our *combination hypothesis*, with reference to university students’ *learning approaches.* In general, there are several research reports that confirm this, in the case of achievement emotions (de la Fuente et al., 2019b), and coping strategies of stress (de la Fuente et al., 2020a). Although learning approaches depend on individual characteristics, they are also fed by characteristics of the teaching process (Howie and Bagnall, 2013), especially in formal teaching-learning contexts at university, an aspect that Biggs (2001) had suggested and which has received consistent empirical support in other recent research reports (Lodewyk et al., 2009; Kember et al., 2020).

#### Effects on Academic Achievement and Satisfaction

Regarding the *second hypothesis*, results allowed us to reject the null hypothesis, since both the established independent variables (SR level and ER level) and their combinations determined levels of total achievement and the sub-types of achievement, as well as determining satisfaction with the teaching-learning experience. This combination effect has already been seen in similar fashion in other previous samples (de la Fuente et al., 2017; Moghimi et al., 2020; Paloş et al., 2020), though the greater effect of the combination on procedural achievement (practical performance subcompetencies: practical problem solving) is a novel finding. By comparison, the greatest effect was seen in total and conceptual achievement, and was determined by regulatory teaching. One plausible explanation for this result is that the regulatory component (times, materiales, learning aids, strategies, meaningful assessment, etc.) is ultimately materialized in better conceptual learning. Notwithstanding, these specific aspects are worthy of further attention and should be clarified in future research.

### Limitations and Future Lines of Research

An initial limitation to this study refers to the sample. Given that the sample is not a heterogeneous group from different disciplines and degree programs, the results should be interpreted with caution. Prior research has shown that whether a teacher’s approach encourages self-regulation, offers external regulation or is lacking in regulation, is dependent on the degree program and the teaching styles of different departments (Kreber et al., 2005; Lindblom-Ylänne et al., 2011). In addition, the concepts of teaching regulation presented in the *Approaches to Teaching Inventory* (Trigwell and Prosser, 2004; Cao et al., 2019) and the concept used in this research on *Regulatory Teaching* are not identical. The former focuses more on an analysis of teaching style, looking at transmission and conceptual change, in order to verify the learning style that it promotes (surface vs. deep approach), while the latter seeks to evaluate whether teaching promotes self-regulation strategies in the students, and thereby affects their learning approach. Future research must accurately establish the relationship between the two concepts of regulation in teaching, as well as the relationship between the teacher’s own self-regulation characteristics and his or her implementation of regulatory teaching (Randi, 2004; Capa-Aydin et al., 2009).

One important limitation of this study is that the assessment system consists exclusively of student self-reports. However, a strength of this study is that both self-assessment (self-regulation, learning approaches, satisfaction) and contextual assessment (regulatory teaching) were included. Nonetheless, future research studies should incorporate complementary assessment systems (Goe et al., 2008; Entwistle and Karagiannopoulou, 2014).

Finally, we are limited in identifying implications for different cultural contexts, because there may be cultural differences in self-regulation, regulatory teaching, and in the relationship between these two variables. Prior research has brought this factor to light, as part of understanding regulation processes (Trommsdorff, 2012; Jaramillo et al., 2017).

### Implications for the Practice of Educational Psychology

These results are of great interest to research and professional practice, allowing us to reconceptualize certain prior evidence and the evaluation of teaching and learning processes at university.

First, there are two important *implications* for *research in this topic*. On the one hand, these consistent and recurring results (some of them reported previously in this Research Topic) indicate the value of analyzing the student’s level of regulation and the level of regulatory teaching in combination, for determining hypothetical levels of cognitive variables, emotional variables, coping and the emotional states of engagement-burnout at university (de la Fuente et al., 2017, 2019b, 2020a, b). These results thus provide empirical support to SRL vs. ERL Theory (de la Fuente, 2017) as a theoretical model for molar analysis, and position the model as a complementary view and a step forward from the SRL model (Zimmerman and Schunk, 2001), taking a more molecular view of analyzing university students’ learning.

In reference to the topic of learning approaches, the present results confirm the strength of this construct, given that they document how learning approaches are sensitive to the effects of the teaching process, which influences the way students pursue their process of learning at university. This idea was already sufficiently recognized in the SAL model, but insufficiently demonstrated in prior research (Biggs, 2001). The prevailing SRL models (see Panadero, 2017) have encouraged research that limits its attention to the student’s intrapersonal variables, leading to large quantities of research production built on the construct of *learning approaches* and its associated inventory (Asikainen and Gijbels, 2017).

The present results, however, encourage us to continue to move forward in integrating both sides into an explanatory analysis of interactive learning behavior, using the proposed combination heuristic.

A second, *practical implication* for applied professional practice has to do with having well-adjusted *conceptions* about how learning approaches are produced in the university context. If we continue to further the idea that learning approaches depend largely on individual variables, to the detriment of context, we will not recognize the important role of the teaching process, just as its authors conceptualized (Biggs, 2001). Without denying the plentiful prior evidence of associated individual characteristics that are determinants of learning approaches, we must progress toward a more interactive, contextualized view of the two processes of learning and teaching (Vermunt, 1998, 2007; Vermunt and Verloop, 1999; Vermunt and Donche, 2017).

A third, *practical implication* refers to *assessing teaching-learning processes* at university, since this is directly related to the issue we have been addressing. Students often participate in assessments of their degree of satisfaction with the teaching process at university, and quality criteria adopted by universities include students’ achievement and their learning approach. If commonly used assessment models continue to focus attention on the teaching process, while overlooking the characteristics of the students who do the assessing, biases are quite likely to exist. Previous research has shown that university students with a surface learning approach, having higher likelihood of poor achievement, tend to give their teachers lower ratings, while students with a deep approach, with greater expectations of success, tend to perceive the teaching process as better in quality (de la Fuente et al., 2011). Furthermore, this assessment practice has another undesirable effect: it is not a contextualized activity for self development, given that students are not assessing themselves with regard to their own characteristics or aspects for improvement in learning, nor with regard to execution of the learning process, but they focus their attention on the teacher and on the teaching process. In this way, students are unlikely to feel that they are equal agents in the process. Using the same logic, teachers likewise are not learning to self-assess their teaching process. For both reasons, it is highly probable that the external attribution of errors and self-attribution of positive aspects adds a bias to this incomplete process.

A final *practical implication* refers to *formative processes* of university teachers (Paris and Winograd, 2003). When implementing innovations in the university teaching process, it is important to consider what type of context is being designed (de la Fuente et al., 2013a). If the context is non-regulating or dysregulating, it will probably not help students improve their learning process, especially if students are low in self-regulation. As seen in prior evidence, students with little self-regulation are the ones that require greater external regulation. Certain prior evidence has shown results that concur with this idea (Shaw et al., 2017; Bingen et al., 2019). In addition, the teacher’s level of self-regulation (Capa-Aydin et al., 2009) increases the likelihood of regulatory teaching (Randi, 2004; Monshi-Toussi et al., 2011), although this relationship has not been addressed in the present study. In an effective teaching process, or regulatory teaching, it is the teacher’s responsibility to design learning environments. To implement such designs, evidence-based recommendations are needed (Roehrig et al., 2012).

## Conclusion

Most universities develop programs –on an intuitive basis– to attract the best students and teachers, based on the correct assumption that a combination of the two produces good learning processes, good academic outcomes, and satisfaction. The practical reality, however, is that different types of students and teachers are found at every university. The present research has offered a *conceptual model*, a *heuristic of measurement*, and consistent *empirical data* for analyzing any teaching-learning process and its most probable effects in a university context, although these can be extrapolated to other stages of education. We must acknowledge that universities admit students who execute “good and not as good” learning processes, and they can be combined with teachers who execute “good and not as good” teaching processes. We recommend that university administrators and organizational politicians, as well as educational psychologists in charge of university quality, take into account the findings presented here, in order to more precisely understand the quality of teaching-learning processes and make appropriate decisions. Not all teachers teach poorly, nor do all students learn well, and viceversa. A detailed analysis of each combination, based on the heuristic presented, should help in making evidence-based decisions in each case (Slavin, 2019).

## Data Availability Statement

The raw data supporting the conclusions of this article will be made available by the authors, without undue reservation.

## Ethics Statement

The studies involving human participants were reviewed and approved by the Comité de Ética de la Investigación (UNAV), ref. 2018.270. The patients/participants provided their written informed consent to participate in this study.

## Author Contributions

JF contributed to conceptualization, design, and data analysis. PS did the initial writing. DK performed the revision of the manuscript. MY did the final revision and adjustments to the manuscript. All authors contributed to the article and approved the submitted version.

## Conflict of Interest

The authors declare that the research was conducted in the absence of any commercial or financial relationships that could be construed as a potential conflict of interest.
